# INVESTIGATING PSYCHOLOGICAL FACTORS AS A PREDICTOR OF FEAR OF FALLING AND FALL RISK IN OLDER ADULTS

**DOI:** 10.1093/geroni/igad104.2563

**Published:** 2023-12-21

**Authors:** Amber Blount, Ladda Thiamwong, Nichole Lighthall, Rui Xie, Joon-Hyuk Park, Victoria Loerzel, Jeffrey Stout

**Affiliations:** University of Central Florida, Orlando, Florida, United States; University of Central Florida, Orlando, Florida, United States; University of Central Florida, Orlando, Florida, United States; University of Central Florida, Orlando, Florida, United States; University of Central Florida, Orlando, Florida, United States; University of Central Florida, Orlando, Florida, United States; University of Central Florida, Orlando, Florida, United States

## Abstract

Psychological factors related to appetitive and avoidant motivation, perceptions of aging, and regulatory focus (promotion focus on gains versus a prevention focus on non-losses) are likely to play an important role in predicting one’s fear of falling (FOF) and resulting impact on physical activity engagement. This pilot study aimed to investigate the gap in current research related to these psychological factors as predictors of older adults’ Fear of Falling and Fall Risk Appraisal (FRA). The study assessed positive and negative perceptions about aging in regards to control and consequences of aging (Brief Aging Perceptions Questionnaire [B-APQ]), motivation (Behavioral Inhibition System/Behavioral Activation system [BIS/BAS]), regulatory focus in goal achievement (Regulatory Focus Questionnaire [RFQ]), FOF (Fall-Efficacy Scale-International [FES-I]), and static balance levels (BTracks Balance system [BBS]). Participants (n=48) were categorized into four FRA groups: rational (low FOF/normal balance), irrational (high FOF/normal balance), incongruent (low FOF/poor balance), and congruent (high FOF/poor balance). A Spearman Rho correlation found that FOF was positively correlated with two of the BAPQ sub-domains of Control-Positive (ρ = 0.46, p<.001) and Consequences and Control Negative (ρ = 0.43, p =.002),. A Kruskal-Wallis test found the relationship between BAPQ Control Positive and the Irrational and Rational FRA group baselines to be significant. The results suggest that among other psychological factors, perceptions about aging may be predictive of FOF and FRA in aging. These results highlight the importance of clinicians addressing not only the physiological factors of fall risk, but also the psychological factors underlying fall risk in older adults.

